# Yellow Fever in New Orleans

**Published:** 1853-11

**Authors:** 


*Yellow Fever in New Orleans.*

The death angel that during the last summer, has hovered over our sister city, gloating its greedy gaze on the victims of the dreadful pestilence, has passed away, restored health has inspired confidence, families are returning to their desolated homes, and the pulse of life beats strongly and firmly, where a few weeks since was the stillness and silence of death. The calamities of this devoted city, have touched the heart of this great nation, stirring its sympathies and inciting to the exercise of a holy God-like charity.

However sad may be this visitation, and however gloomy may be the shadow which it has cast over our whole country, it has still furnished the occasion for the exercise of those noble ornaments of our natures, *brotherly love and relief.* The members of the Howard Association, by their assiduous care for the sick, and by the exposure of their own persons, to the deadly infection in endeavoring to alleviate the sufferings of the dying, have illustrated a name which will be ever held sacred by humanity.

The clergy with but few exceptions, remained with their flocks, administering in the hour of trial, the consolations of religion, pouring into the wounded heart the balm of sympathy, and inspiring it with the hope of a brighter, purer and holier world, where suffering is unknown.

But physicians, more than any other class of men, have been re quired to perform a herculean labor, and to feel the most intense and painful anxiety. During the last few months 20,000 *poor* persons sick of the epidemic, have been treated by the members of the medical profession in New Orleans. More than three-fourths of the whole medical services, rendered during the last summer have been gratuitous. The cooling breath of evening, at the close of the weary day has brought to them no assurance of rest, for the pestilence slumbereth not. In each new victim, death and the physicians met and measured swords, and cases were not unfrequent in which, worn with fatigue and disheartend at the fearful sights which they were called to witness, they returned to their homes only to lie down and die.

We might reasonably expect, in view of the sacrifices which the profession has made, that its services would be acknowledged, and such we believe in the majority of instances is the case.

We cannot forbear however, in this connection, from alluding to a discourse recently delivered in this city, by the talented editor of the *North Western Christian Advocate and Journal*, on the pestilence in New Orleans. The Howard Association, and the clergy of that afflicted city, were alluded to in terms honorable alike to the head and heart of the speaker, while a single reference was made to the members of the medical profession in which they were sneeringly called the " Sapient sons of Esculapius." We respect the man, but we despise the bigotry that ignores merit, because it happens to be found without the circle of the "holiest men in the world." We seek no favors; we ask no gratuitous services of the public, or at the hands of the clergy; but we demand for ourselves and for our brethren in New Orleans, simple justice. J.

